# Antimicrobial activities of endophytic fungi isolated from *Ophiopogon japonicus* (Liliaceae)

**DOI:** 10.1186/1472-6882-12-238

**Published:** 2012-11-28

**Authors:** Hanqiao Liang, Yongmei Xing, Juan Chen, Dawei Zhang, Shunxing Guo, Chunlan Wang

**Affiliations:** 1 The Key Laboratory of Bioactive Substances and Resource Utilization of Chinese Herbal Medicine, Ministry of Education, Institute of Medicinal Plant Development, Chinese Academy of Medical Sciences & Peking Union Medical College, Beijing, 100193, Peoples Republic of China

**Keywords:** *Ophiopogon japonicus*, Endophytic fungi, Peptide deformylase, Antimicrobial activity

## Abstract

**Background:**

Drug resistance in bacteria has become a global concern and the search for new antibacterial agents is urgent and ongoing. Endophytes provide an abundant reservoir of bioactive metabolites for medicinal exploitation, and an increasing number of novel compounds are being isolated from endophytic fungi. *Ophiopogon japonicus*, containing compounds with antibacterial activity, is a traditional Chinese medicinal plant used for eliminating phlegm, relieving coughs, latent heat in the lungs, and alleviating diabetes mellitus. We investigated the antimicrobial activities of 30 strains of *O. japonicus*.

**Methods:**

Fungal endophytes were isolated from roots and stems of *O. japonicus* collected from Chongqing City, southwestern China. Mycelial extracts (MC) and fermentation broth (FB) were tested for antimicrobial activity using peptide deformylase (PDF) inhibition fluorescence assays and MTT cell proliferation assays.

**Results:**

A total of 30 endophytic strains were isolated from *O. japonicus*; 22 from roots and eight from stems. 53.33% of the mycelial extracts (MC) and 33.33% of the fermentation broths (FB) displayed potent inhibition of PDF. 80% of MC and 33.33% of FB significantly inhibited *Staphylococcus aureus*. 70% of MC and 36.67% of FB showed strong activities against *Cryptococcus neoformans*. None showed influence on *Escherichia coli*.

**Conclusion:**

The secondary metabolites of endophytic fungi from *O. japonicus* are potential antimicrobial agents.

## Background

Endophytes are microorganisms that live in the intercellular spaces of healthy host tissues without causing obvious symptoms [1]. An increasing number of compounds with antibacterial activity are being isolated from endophytic fungi, including fumitremorgins B isolated from *Phomopsis* sp., and periconicins A and B from *Periconia* sp. [2,3]. Exploitation of novel classes of antimicrobial metabolites is increasingly noticeable over recent years. A considerable body of research has investigated the diversity, ecological role, secondary metabolites and bioactivity of the endophytic fungi isolated from various medicinal plants [4].

The antibiotic resistance of bacterial pathogens has become a serious health concern and encourages the search for novel and efficient antimicrobial metabolites. There has therefore been a tremendous increase in interest in screening endophytes for their antimicrobial activities. Approximately 50 species belong to the genus *Ophiopogon* (Liliaceae) and most are found in eastern and southern Asia. Thirty-three *Ophiopogon* species and four varieties are available in China [5]. *O. japonicus* is a traditional medicine, admitted as one of functional food ingredient by the Ministry of Health of the People’s Republic of China. Over thousands of years, *O. japonicus* has been used to relieve symptoms such as coughing, phlegm, and heat in the lungs caused by bacterial infection [6-9]. Research has shown that the ‘Compound *Ophiopogon japonicus* Pill’ has displayed effective inhibition of *Staphylococcus aureus* and an extract from *O. japonicus* showed strong inhibition of malic mildew [10,11]. Furthermore, previous studies have shown that *O. japonicus* inhibited germination and growth of several bacteria and fungi [12-14]. Previous research showed that ruscogenin from *O. japonicus* displayed remarkable antibacterial activity [15,16], while the homoisoflavonoids from *O. japonicus* showed significant suppressive effects on eotaxin expression induced by IL-4 [17]. To the best of our knowledge, certain endophytic fungi can produce similar or the same active metabolites as their host plants [18]. We therefore think it necessary and timely to study the antimicrobial activities of the fungal endophytes associated with *O. japonicus*.

Our study was undertaken to screen endophytic fungi for activities against microorganisms and peptide deformylase (PDF), an essential enzyme in bacterial protein biosynthesis and shown to be an exciting target for the discovery of novel and efficient antibiotics. The antibacterial spectrum of PDF inhibitors currently published is primarily gram positive, including *Staphylococci* and *Streptococci*, and PDF inhibitors show no cross-resistance with existing antibiotic classes [19]. However, only limited information about endophytes from *O. japonicus* and their antibacterial activities has been gained to date. Screening of endophytic fungi from *O. japonicus* for antimicrobial activities will serve as a good basis for discovering new antibiotic agents.

## Methods

### Isolation and identification of endophytic fungi

Healthy roots and stems of *O. japonicus* were collected from Chongqing City, southwestern China. The host plant was identified by Professor Li Biao of the Institute of Medicinal Plant Development (IMPLAD), of the Chinese Academy of Medical Sciences, and deposited in the Herbarium of the Department of IMPLAD, Beijing, China. Fungal isolation was carried out following Arnold [20], but with minor modifications. Samples were rinsed with running water and processed as follows. Samples were immersed in 75% ethanol for 1 min and in NaOCl (3%) for 5 min, washed three times with sterile distilled water, and then allowed to surface-dry on sterilized filter paper. Finally, samples were cut into 0.5 × 0.5 cm pieces and placed in Petri dishes (9 cm in diameter) on potato dextrose agar (PDA) medium (g/l; dextrose-20, agar-15, potato infusion-200), and cultured at 25°C in the dark for 1–2 weeks [21]. Active endophytic fungi were identified morphologically and by molecular analysis of the internal transcribed region (ITS) of the ribosomal DNA as described by Chen [22].

### Preparation of crude fungal extracts

Crude extracts of endophytic fungi were prepared as described by Wang [23], but with slight modifications. Endophytic cultures were filtered using nylon nets to separate the culture broth and mycelia. All filtrates were transferred to larger conical flasks filled with five times the volume of 95% ethanol, stirred fully and left overnight, and then further concentrated in a vacuum to remove organic solvents. Mycelia were extracted twice with ultrasonic waves and again evaporated to dryness. EtOH extracts were dried by freeze drying, and then diluted with sterile distilled water to a concentration of 10 mg/ml and sterilized by filtration through a 0.22 μm millipore filter for antimicrobial activity assay. Extracts were dissolved in 1 ml dimethyl sulfoxide (DMSO) and kept at 4°C for PDF inhibitory activity assay.

### Peptide deformylase inhibitory fluorescence assay

The PDF gene was cloned and the product purified as previously described by the endpoint method [24-30]. Briefly, total DNA was extracted from cells of *E. faecium* ATCC6057 and the PDF gene was amplified by PCR using the primer pairs 5^′^-GGGAATTCCATATGATTACAATGG-3^′^ and 5^′^-CCGCTCGAGCTACTCGATCACC-3^′^. The gene was then cloned into the pET28a (+) vector and expressed in *E. coli* BL21 (DE3). The PDF enzyme was purified with a Ni-NTA Superflow Column (Qiagen) using sodium phosphate buffer and an imidazole gradient. Purified enzyme and substrate (For-Met-Ala-Ser, Bachem) were mixed and then incubated with samples for 1 h at 37°C in 50 μl reaction volumes. Finally, 2 μl of a fluorescamine (Sigma) solution (2 μg/ml in DMSO) was added and then the fluorescence was determined with the excitation wavelength at 390 nm and the emission wavelength at 490 nm using a fluorescamine plate reader (Perkin Elmer).

### *In vitro* antimicrobial activity

The crude extracts from the endophytes of *O. japonicus* were tested against one gram-positive bacterium (*S. aureus*), one gram-negative bacterium (*E. coli*), and one pathogenic fungus (*C. neoformans*), using a concentration of 1000 μg/ml. All indicator bacteria and fungi were obtained from the China General Microbiological Culture Collection Center (CGMCC) in Beijing, China. We tested the antimicrobial activity by MTT 3-(4,5-dimethyl thiazol-2-yl)-2,5-diphenyl tetrazolium bromide colorimetric assay (MCA), as previously described [31,32], but with a slight modification. Briefly, 80 μl of the indicator strain suspension with a density of 1 × 10^6^ CFU/ml and 10 μl of sample were added into each well of a 96 well flat-bottom microtiter plate, and then incubated for 24 h/48 h at 37°C/28°C. After that, 10 μl of 5 mg/ml MTT solution was added into each well. Four hours later the cultures were centrifuged to precipitate formazan crystals, 200 μl of dimethylsulfoxide (DMSO) was added, and then the cultures were incubated for 10 min at room temperature after supernatants were removed [33]. Finally, the optical density (OD) of the formazan solution was read at the wavelength of 570 nm, with 690 nm as a reference read-out. For every experiment, a background control, negative control, and positive control (gentamycin sulfate for bacteria, fluconazole for fungi) were included [34]. Minimum inhibitory concentration (MIC) was assigned to the lowest concentration for which no blue appeared.

## Results and discussion

Of the total 30 endophytes obtained from *O. japonicus*, 22 endophytes (73.3%) were isolated from roots, and the remainder from stems. Endophytic fungi from roots were thus much more common than those from stems. The possible reason for this is that endophytic fungi are heterotrophic eucaryons. The roots play a very important role in synthesizing and storing of nutritive matter which will promote the metabolism, communication, and transport of nutrients in which the endophytes are involved.

**Figure 1:**
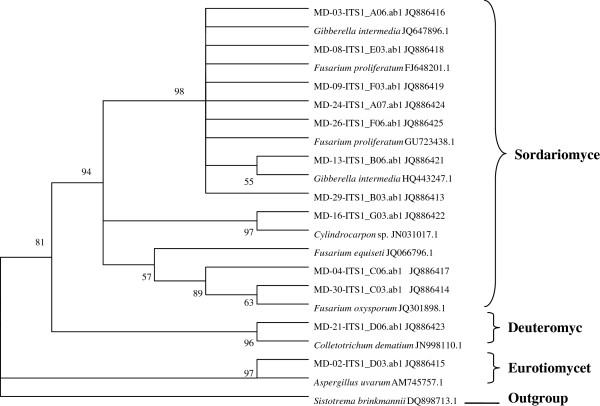
**Neighbour-joining (NJ) phylogenetic tree based on ITS-rDNA sequences of endophytic fungi associated with the *Ophiopogon japonicus*.** Numbers above or below branches indicate bootstrap values of NJ and MP analyses (> 50%, right) from 1000 bootstrap replicates.

**Figure 2:**
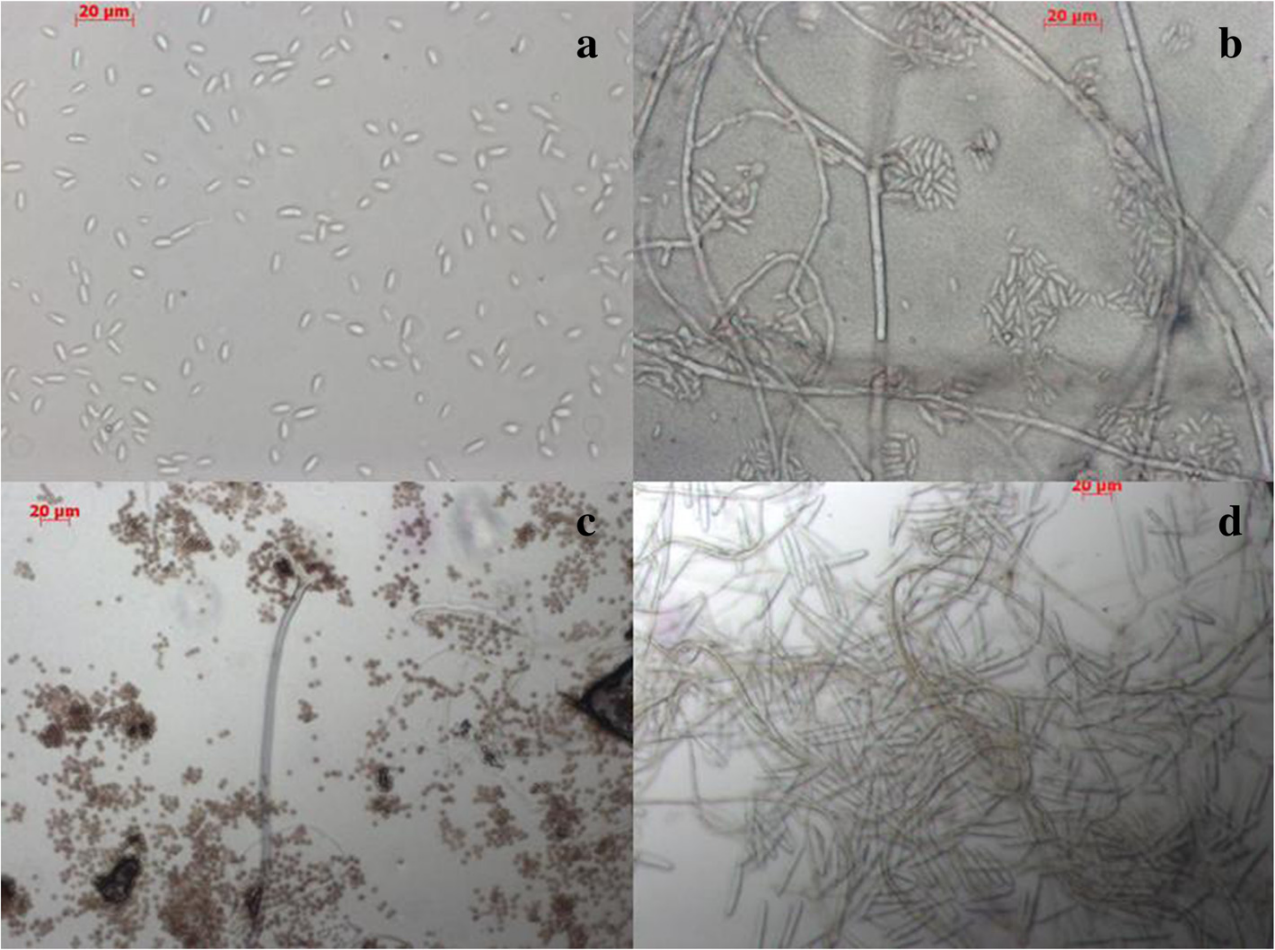
**Light micrographs of endophytic fungi isolated from *O. japonicus*. a** and **b***Fusarium* isolated from stem and root of *O. japonicus*. **c***Aspergillus* isolated from root of *O. japonicus*. **d***Cylindrocarpon* isolated from root of *O. japonicus.*

Our phylogenetic analysis indicated 12 active strains belonging to the phylum Ascomycota, and able to be classified into three taxonomic classes (Sordariomycetes, Deuteromycetes, and Eurotiomycetes). From morphology and ITS analysis we determined that these 12 active strains belonged to four genera (Figures 1 and 2).

Twelve isolates showed the inhibition of PDF enzyme levels, while showing resistance to *S. aureus* at the cellular level. Among the active isolates, *Fusarium* was the predominant genus with about 75% of strains, but the different isolates in this genus showed different strengths of antimicrobial activity. This is consistent with previous research [35-38]. All the antimicrobial activities of the extracts are shown in Table 1. We observed that crude extract of FBs from stems mostly displayed inhibitory activity on PDF, while the same extract showed no activity against *S. aureus*; illustrating that the extracts do not penetrate cell membranes.

**Table 1:** **Antimicrobial activities and culture time of endophytic fungi isolated from *O. japonicus***

**Strain no.**	**Position**	**Inhibition rate of PDF**		**Inhibition rate of antimicrobial activity**						**Incubation time (day)**
				***S.aureus***		***E. coli***		***C. neoformans***		
		**MC**	**FB**	**MC**	**FB**	**MC**	**FB**	**MC**	**FB**	
MD-01	Root	—	—	+	—	—	—	—	—	4
MD-02	Root	++	—	+	—	—	—	—	+	4
MD-03	Root	—	—	+++	—	—	—	+	—	14
MD-04	Root	++	—	++	++	—	—	—	—	11
MD-05	Root	+	—	—	+	—	—	—	—	9
MD-06	Root	—	++	+	—	—	—	+	—	10
MD-07	Root	—	—	+	+	—	—	—	—	6
MD-08	Root	++	—	++	+	—	—	—	—	15
MD-09	Root	+++	—	+++	+++	—	—	+	+++	14
MD-10	Root	+++	—	—	+	—	—	+	—	9
MD-11	Root	++	—	—	—	—	—	—	+	9
MD-12	Root	+++	—	—	—	—	—	++	++	5
MD-13	Root	+++	—	+	—	—	—	—	—	6
MD-14	Root	—	—	+	—	—	—	+	—	10
MD-15	Root	—	—	+++	—	—	—	++	—	5
MD-16	Root	—	—	++	—	—	—	+	+	5
MD-17	Root	—	—	—	—	—	—	+	—	10
MD-18	Root	++	—	+	—	—	—	++	—	10
MD-19	Root	—	—	++	—	—	—	++	+	6
MD-20	Root	—	—	+	—	—	—	+	—	16
MD-21	Stem	+	+	+	—	—	—	++	—	6
MD-22	Stem	—	++	+	—	—	—	+	—	5
MD-23	Stem	—	+	+	—	—	—	++	—	4
MD-24	Stem	+	++	++	++	—	—	+	++	14
MD-25	Stem	—	++	+	—	—	—	++	+	4
MD-26	Stem	++	+	+	++	—	—	+	+	14
MD-27	Stem	—	+++	+	—	—	—	++	—	4
MD-28	Stem	+	+++	—	—	—	—	+	+	4
MD-29	Root	++	—	++	+++	—	—	—	++	10
MD-30	Root	+	++	++	+++	—	—	+	+	10

The peptide deformylase inhibition and antimicrobial activities are expressed by the inhibition rate (IR): -, inhibition rate <30%; +, 30% ≤ inhibition rate <60; ++, 60% ≤ inhibition rate <90%; +++, 90 ≤ inhibition rate.

We found that the activities of MCs were better than FBs. However, the FB of strain MD-09, a *Gibberella* sp., that exhibited the greatest inhibition *S. aureus* and *C. neoformans* (MIC = 20 μg/ml, 80 μg/ml), also exhibited significant activities on PDF. In addition, the MC of MD-09, MD-10, MD-12, MD-30, and the FB of MD-27 and MD-28 showed significantly inhibition of PDF (IR > 90%); the MC of MD-03, MD-09, MD-15, and the FB of MD-09, MD-29, and MD-30 inhibited more than 90% growth of *S. aureus* (MIC 20–40 μg/ml), while only the FB of MD-09 showed strong inhibition against *C. neoformans*. It thus appears that the MD-09 strain could be a source of bioactive antibacterial agents, and its metabolites are worth further research.

No extract displayed activity against *E. coil*. This is not consistent with previous research by Li [39], who studied the fungistatic and promoter action of *O. japonicus*, and found light concentration against *E. coil* while high concentration accreting growth of *E. coil*. The underlying reasons for this incompatibility are worth further investigation. We also screened for inhibition of HIV-1 integrase, but without obvious activity.

In addition, the crude extracts of *Fusarium oxysporum* and *Fusarium poae*, isolated from *O. japonicus*, were investigated for their prominent inhibition of several phytopathogens [40]. Many studies have indicated that *Fusarium* sp. are the most common species and a potent source of bioactive compounds among endophytes from medicinal plants. Previous research has shown the abundance of secondary metabolites of *Fusarium* that have antimicrobial activity. The pentaketide (CR377: 2-methylbutyraldehyde-substituted-α-pyrone) from a *Fusarium* sp. (in *Selaginella pallescens*) showed strong activity against *Candida albicans*[41]. A *Fusarium* sp. from *Tripterygium wilfordii* produced antimicrobial compounds such as subglutinol A and B [42]. In addition, beauvericin from *Fusarium oxysporum* isolated from the bark of *Cinnamomum kanehirae* significantly suppressed growth of methicillin-resistant *S. aureus* and *Bacillus subtilis*[43].

Furthermore, antimicrobial metabolites produced by the fermentation of endophytic fungi have many advantages, including no destruction of resources, sustainable use, easy large-scale industrial production and quality control. Our research supports the previous finding that endophytic fungi display high antimicrobial activities. We plan to research and develop new antibiotics based on our present findings.

## Conclusions

The preliminary results of screening endophytic fungi from *O. japonicus* indicate their potential generation of bioactive metabolites for novel antibiotic discovery. Strain MD-09 displayed significant activity, revealing its potential for development as an antimicrobial drug, and clearly deserving further research.

## Abbreviations

MC: Mycelium; FB: Fermentation broth; IR: Inhibition rate; PDF: Peptide deformylase; OD: Optical density; PDA: Potato dextrose agar.

## Competing interests

The authors declare that they have no competing interests.

## Authors’ contributions

HQL was the principal investigator who designed the study, isolated the endophytic fungi, and wrote the manuscript. YMX participated in the design of the *in vitro* antimicrobial activity study and in writing the manuscript. JC carried out the the model of peptide deformylase inhibition fluorescence assay. DWZ carried out the development screening model for inhibitors targeting strand transfer reaction of HIV-1 integrase. SXG and CLW participated in the design of the study, revised the manuscript, and provided financial support. All authors read and approved the final manuscript.

## Pre-publication history

The pre-publication history for this paper can be accessed here:

http://www.biomedcentral.com/1472-6882/12/238/prepub
